# Impact of using a perioperative artificial endocrine pancreas in pancreatic resection

**DOI:** 10.1002/ags3.12374

**Published:** 2020-07-18

**Authors:** Toshiaki Yoshimoto, Tetsuya Ikemoto, Yuji Morine, Satoru Imura, Yu Saito, Shinichiro Yamada, Katsuki Miyazaki, Yukako Takehara, Mitsuo Shimada

**Affiliations:** ^1^ Department of Surgery Tokushima University Tokushima Japan

**Keywords:** artificial pancreas, complications, glycemic control, lymphocyte, pancreatectomy

## Abstract

**Aim:**

Pancreatectomy causes both hyperglycemia, secondary to surgical stress, and pancreatic diabetes, which leads to difficult‐to‐control postoperative blood glucose levels. We investigated whether using an artificial pancreas perioperatively to provide appropriate blood glucose control could reduce postoperative complications following pancreatectomy.

**Methods:**

We retrospectively enrolled 52 patients who underwent pancreatectomy at Tokushima University Hospital from 2015 to 2019. The most recent 26/52 patients received perioperative blood glucose control using an artificial pancreas. Postoperative blood glucose control with manual insulin injections based on a sliding scale was performed in the earlier 26 patients (controls). We compared surgical outcomes between the artificial pancreas group and the control group.

**Results:**

There was no significant difference in patients' white blood cell or neutrophil counts, prognostic nutritional index, neutrophil‐lymphocyte ratio, and C‐reactive protein‐to‐albumin ratio on postoperative day 1; however, lymphocyte counts were higher in the artificial pancreas group. The number of serious complications of Clavien‐Dindo grade >IIIa was significantly lower in the artificial pancreas group (*P* < .05).

**Conclusions:**

Using an artificial pancreas for perioperative blood glucose control in patients undergoing pancreatectomy decreased the number of serious complications through proper management of blood glucose levels without hypoglycemia, and may influence peripheral lymphocytes.

## Graphical abstract

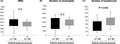

## 1 INTRODUCTION

Surgical operations exert considerable physical stress on patients perioperatively, and recent studies demonstrated that hyperglycemia exacerbates the inflammatory response and leads to oxidative stress, poor immune function, and endothelial dysfunction.^1^, ^2^ Hyperglycemia is a risk factor for infectious diseases and results in increased comorbidities and mortality, and maintenance of blood glucose levels at <180 mg/dL has been recommended.^3^ A previous retrospective study reported that in a comparison of intensive care patients in survival and nonsurvival groups, the survival group demonstrated significantly less blood glucose level variability.^4^ In 2003, a randomized controlled trial indicated that strict control of blood glucose with insulin in a surgical intensive care unit reduced postoperative morbidity and mortality, and that subsequent organ failure could be ameliorated and survival rates improved by maintaining blood glucose levels between 80 and 110 mg/dL.^5^ However, studies also reported that intensive insulin therapy (IIT) was associated with hypoglycemic attacks^6^ and that hypoglycemia with blood glucose levels < 40 mg/dL or levels of 70 mg/dL associated with conventional glycemic control methods, such as tight glycemic control with an open‐loop system and the sliding‐scale method, can cause fatal complications in the presence of neurological disorders.^7^ Therefore, blood glucose management by closed‐loop systems is currently attracting attention.

An artificial pancreas has recently been developed that stabilizes glucose levels by continuously administering insulin and monitoring blood glucose. One study reported that glucose control using an artificial pancreas was stricter than that using the sliding scale method.^8^ Perioperative tight glycemic control using a bedside artificial pancreas with a closed‐loop system has also been proven safe and effective for avoiding hypoglycemia as well as for reducing blood glucose level variability, and resulted in good surgical outcomes.^9^ The use of an artificial pancreas for perioperative blood glucose management in a patient with glycogen storage disease or critically ill patients, such as those with severe burns, was beneficial in managing blood glucose levels without hypoglycemia.^10^, ^11^

In hepatobiliary pancreatic surgery, diabetes associated with pancreatectomy is termed pancreatogenic diabetes. This complication necessitates high amounts of insulin and is associated with difficult‐to‐control postoperative blood glucose levels after pancreatic surgery.^12^, ^13^ Furthermore, the surgery itself is invasive and the incidence of complications is high.^14^ In this study, we evaluated whether using an artificial pancreas could reduce postoperative complications following pancreatectomy.

## 2 MATERIALS AND METHODS

### 2.1 Patients and methods

This study was performed in accordance with the Helsinki Declaration of the World Medical Association. Our hospital introduced the use of an artificial pancreas in hepato‐biliary‐pancreatic surgeries in 2019. This retrospective study enrolled 52 patients who underwent pancreatectomy at Tokushima University Hospital from 2015 to 2019. All patients provided written informed consent, and the study protocol was approved by the institutional review board of Tokushima University Hospital (approval number from the Tokushima Clinical Trial Management System [ToCMS]: 3215). Of the 52 patients, perioperative blood glucose control using an artificial pancreas was performed in 26 patients since 2019 (artificial pancreas group). Postoperative blood glucose control with manual insulin injections based on commonly used sliding scales was performed in the previous 26 patients (control group).

Glucose concentrations were controlled with a programmed infusion of insulin determined by the control algorithm of the STG‐55 artificial pancreas (Nikkiso Inc) in the artificial pancreas group. The target blood glucose level was 120‐180 mg/dL. Strict blood glucose control using the artificial pancreas was performed from the beginning of the operation to the next morning in the intensive care unit. Thereafter, the same postoperative management was performed for both groups, according to the clinical guidelines of our hospital.

In all cases, intravenous hyperalimentation (IVH) was used immediately after surgery, and cefmetazole was used as the antibacterial drug according to the clinical path.

### 2.2 Parameters

We recorded the following patient clinicopathological data: age, sex, body mass index, diabetes mellitus, hemoglobin A1c, postoperative weight loss, white blood cell count and its fraction on postoperative day 1, prognostic nutritional index (PNI), neutrophil‐lymphocyte ratio (NLR), C‐reactive protein to albumin ratio (CAR), and postoperative complications.

### 2.3 Statistical analysis

Univariate analysis of the differences between the groups was determined by log‐rank tests, and multivariate analysis by chi‐squared tests. All statistical analyses were performed using JMP version 13.0 statistical software (SAS Institute Inc). Two‐sided *P* values < .05 were considered significant.

## 3 RESULTS

### 3.1 Patient characteristics

Table 1 shows the pancreatic disease and surgical procedures of the patients who underwent pancreatectomy in this study. The 52 patients constituted 28 men and 10 women with an average age of 63.6 ± 1.9 years (range, 33‐84 years). Twenty‐four patients (46%) were diagnosed with type 2 diabetes mellitus before surgery. Of the 26 patients managed with the artificial pancreas, 14 patients (53.8%) underwent subtotal stomach‐preserving pancreaticoduodenectomy, 10 (38.5%) underwent distal pancreatectomy, and one (3.8%) underwent total pancreatectomy. Of the 26 patients treated with insulin injections using a sliding scale, 13 patients (50.0%) underwent subtotal stomach‐preserving pancreaticoduodenectomy, 10 (38.5%) underwent distal pancreatectomy, and two (7.7%) underwent total pancreatectomy. There was no significant difference in the number of patients undergoing the different surgical procedures between the two groups.

**TABLE 1:** Pancreatic disease and surgical procedures performed for the 52 patients undergoing pancreatectomy

	All cases (n = 52)	Control (n = 26)	Artificial pancreas (n = 26)
Disease			
Pancreatic cancer	40	23	17
Biliary cancer	6	2	4
IPMN	4	1	3
MCN	1	0	1
SCN	1	0	1
Procedures			
SSPPD	27	13	14
DP	20	10	10
TP	3	2	1
Subtotal pancreatectomy	1	1	0
Total remnant pancreatectomy	1	0	1

Abbreviations: DP, distal pancreatectomy; IPMN, intraductal papillary mucinous neoplasm; MCN, mucinous cystic neoplasm; SCN, serous cystic neoplasm; SSPPD, subtotal stomach‐preserving pancreaticoduodenectomy; TP, total pancreatectomy.

### 3.2 Blood glucose control in the intraoperative and perioperative period

Table 2 shows the intraoperative and perioperative blood glucose levels in the patients of both groups. In the artificial pancreas group, stable glycemic control was achieved within the target blood glucose range with mean blood glucose levels of 144.3 ± 21.8 mg/dL. The average minimum blood glucose level was 98 ± 13.4 mg/dL, and no severe hypoglycemia was observed. The period of management using the artificial pancreas was 1361.2 ± 331.0 minutes. On the other hand, control group showed significantly higher blood glucose levels than the artificial pancreas group, both at the mean and at the highest and lowest value. More notably, there was significant hypoglycemia of under 60 mg/dL in control group, and it is possible that there was actually more severe hypoglycemia.

**TABLE 2:** Perioperative blood glucose changes in patients with or without artificial pancreas

Blood glucose (mg/dL)	Control (n = 26)	Artificial Pancreas (n = 26)	*P* value
Average^a^	184.2 ± 28.2 (129.6‐240.8)	144.3 ± 21.8 (85.1‐167.5)	<.001
Maximum^b^	258.5 ± 47.3 (182.0‐361.0)	233.8 ± 23.0 (181.5‐284.1)	.021
Minimum^c^	117.8 ± 22.5 (58.0‐170.0)	98.2 ± 13.4 (72.4‐126.5)	<.001

^a^ Average of the blood glucose level from the beginning of the operation to the next morning in the intensive care unit (perioperative period).

^b^ Maximum values of blood glucose in perioperative period.

^c^ Minimum values of blood glucose in perioperative period. Each data showed average ± standard deviation (minimum – maximum value).

### 3.3 Preoperative patient backgrounds

Table 3 shows the preoperative backgrounds of the patients and postoperative outcomes. There was no significant difference in the patients' preoperative backgrounds between the control group and the artificial pancreas group, and type 2 diabetes mellitus (DM) was observed in 11 and 13 patients (42% and 50%), respectively. There were no differences of preoperative insulin dose in DM patients, blood cell count, and CRP level between two groups.

**TABLE 3:** A comparison of the characteristics and data from patients in the control and artificial pancreas groups undergoing pancreatectomy

Factors		Control (n = 26)	Artificial Pancreas (n = 26)	*P* value
Age	Mean	66.2 ± 7.3	71.2 ± 10.4	.073
Sex	M/ F	14/ 12	13/ 13	.781
BMI	Mean	20.7 ± 3.1	22.5 ± 2.9	.043
DM	‐/ +	15/ 11	13/ 13	.578
HbA1c	Mean	6.27 ± 1.13	6.68 ± 1.91	.363
Pancreatic hardness	Soft/ Hard	18/ 6	18/ 6	1
Pancreatic duct diameter (mm)	Mean	5.04 ± 0.66	3.92 ± 0.32	.136
Post‐operative data of day 1				
WBC	<15 000/≥15 000	8/ 18	12/14	.254
Neutro	<15 000/≥15 000	5/ 5	12/9	.709
Lymph	<500/≥500	6/ 4	4/17	.023
Alb	<2.5/≥2.5	13/13	12/14	.781
CRP	<10/≥10	12/14	9/17	.397
PNI	<30/≥30	7/3	17/ 9	.793
NLR	<20/≥20	4/6	12/10	.447
CAR	<5/≥5	14/12	15/11	.780
Post‐operative complications	‐/+	9/17	15/11	.095
CD≥IIIa	‐/+	18/8	24/2	.035
SSI	‐/+	25/1	25/1	1.000
POPF	‐/+	17/9	21/5	.211
Bil. fistula	‐/+	24/2	26/0	.149
Chylorrhea	‐/+	23/3	26/0	.074
Post‐operative weight loss	<15%/≥15%	22/4	23/1	.187
Post‐operative hospital stay (days)	Mean	25.1 ± 9.9	21.9 ± 10.6	.272

Abbreviations: Alb, albumin; Bil., biliary; BMI, body mass index; CAR, C‐reactive protein‐to‐albumin ratio; **C**D, Clavien–Dindo classification; CRP, C‐reactive protein; DM, diabetes mellitus; HbA1c, hemoglobin A1c; Lymph, lymphocyte count; Neutro, neutrophil count; NLR, neutrophil–lymphocyte ratio; PNI, prognostic nutritional index; POPF, Postoperative pancreatic fistula; SSI, surgical site infection; WBC, white blood cell.

### 3.4 Postoperative outcomes

**FIGURE 1:**
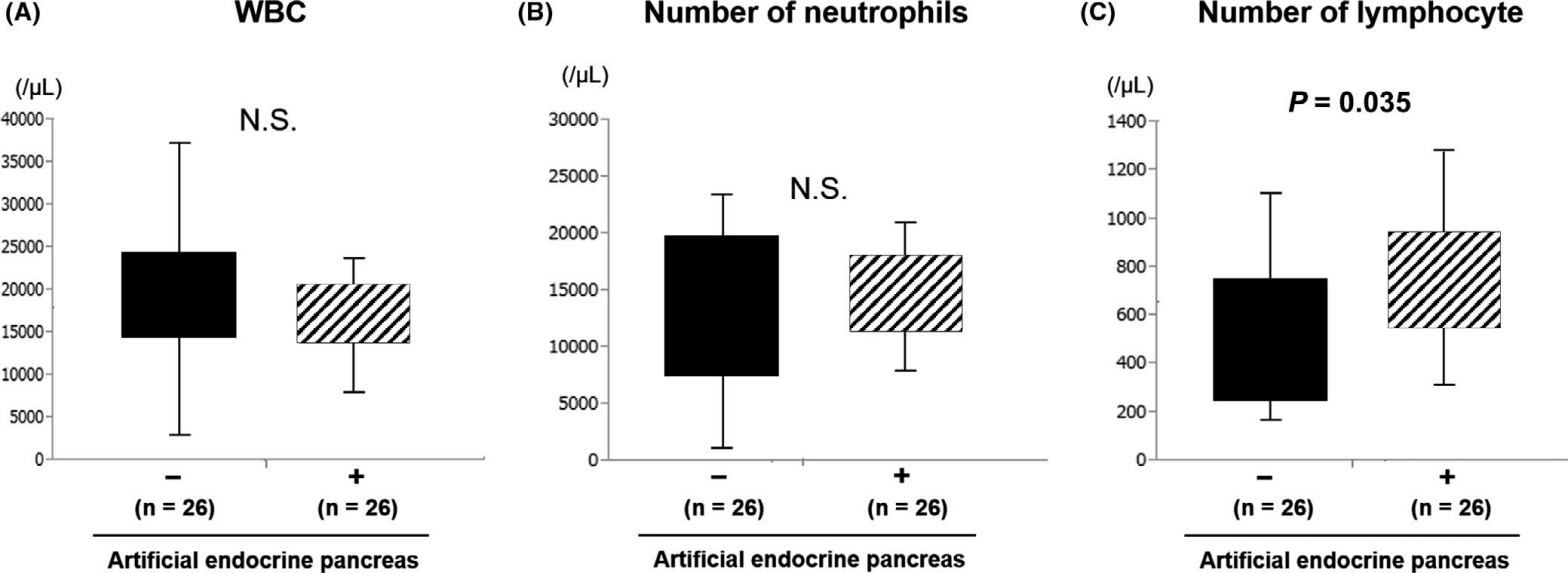
Change in blood count before and after surgery. There was no significant difference in white blood cell and neutrophil counts on postoperative day 1. Lymphocyte counts were higher in the artificial pancreas group

**FIGURE 2:**
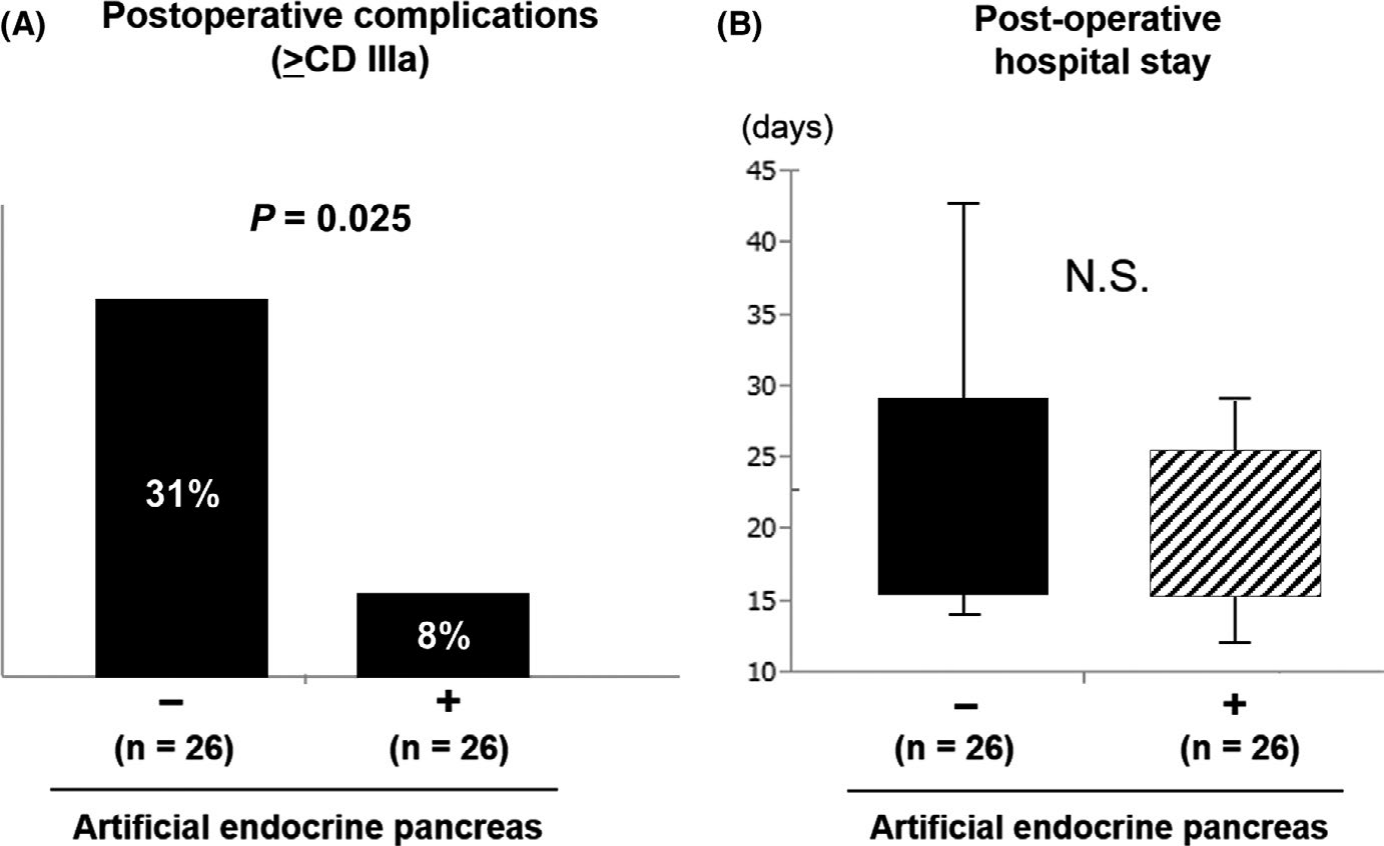
Postoperative outcomes. A, The number of serious complications > Clavien–Dindo grade IIIa was significantly lower in the artificial pancreas group. B, There was no significant difference in the length of hospital stay after surgery

There was no significant difference in patients' white blood cell and neutrophil counts on postoperative day 1, but lymphocyte counts were higher in the artificial pancreas group (*P* < .05) (Figure 1). Regarding the nutritional evaluation indices, there was no significant difference for PNI, NLR, and CAR. Regarding postoperative complications, the number of serious complications Clavien‐Dindo grade (CD) >IIIa was significantly lower in the artificial pancreas group (*P* < .05) (Figure 2A); however, there was no significant difference in the length of hospital stay after surgery (Figure 2B). In the control group, CD III or higher complications included six cases of postoperative pancreatic fistula (POPF), one case of biliary fistula, and one case of intraperitoneal hemorrhage from the mesenteric artery and this patient needed open hemostasis. In the artificial pancreas group, CD IIIa complications were two cases of POPF and no patient suffered CD IIIb or higher complications. All patients with CD IIIa complications required either drain replacement or insertion. The mortality rate was 0% in both groups.

## 4 DISCUSSION

Hyperglycemia induced by surgical stress often dysregulates liver metabolism and immune function, resulting in impaired postoperative recovery.^12^ Moreover, perioperative hyperglycemia also plays a significant role in the development of postoperative infection.^15^ As a countermeasure, IIT is performed for critically ill patients, and its specific aims are multiple organ protection and the prevention and treatment of infection by normalizing and maintaining blood glucose concentrations at normal levels.^16^ However, it is unclear how soon blood glucose falls after insulin administration, and it has recently become clear that hypoglycemia has a greater prognostic effect.^15^ To address the problem of hypoglycemia, an artificial pancreas has recently been developed that stabilizes glucose levels by continuously administering insulin and monitoring blood glucose.^1^ Using this system, strict glycemic control approaching normoglycemia was achieved, such as targeting blood glucose in the range of 80‐110 mg/dL, without hypoglycemia, and with less variability in blood glucose concentration.^7^ A randomized clinical trial showed that tight perioperative glycemic control using a closed‐loop artificial pancreas system decreased the rate of surgical site infections (SSI) in patients who underwent pancreatectomy or hepatectomy.^17^

In our study, there was no significant difference in the rate of occurrence of SSI; however, although the number of patients in this study was relatively low, serious complications over CD IIIa were significantly reduced in the artificial pancreas group. According to the clinical path, the drain is removed by postoperative day 7 without POPF, bile leakage or deep SSI. In this study, the overall incidence of POPF or biliary fistula did not change, however, the deep SSI of CDIIIa, which required drain replacement, decreased in the artificial pancreas group. There were no significant differences in the nutritional evaluation indices PNI, NLR, and CAR; however, lymphocyte counts on postoperative day 1 were higher in the artificial pancreas group. Although there is no doubt that perioperative nutritional management is important in pancreatectomy,^18^ one study reported that nutritional assessment scores do not contribute to mortality and morbidity.^19^ In contrast, lymphocyte counts often decrease after major surgical operations and are considered to reflect an immunosuppressed state.^20^ Previous reports showed that the number of lymphocytes decreased significantly only 2 hours after transient hyperglycemia.^21^ In addition, it is reported that there is a correlation between postoperative lymphocyte count reduction and the incidence of postoperative pneumonia after surgery for lung cancer.^22^ We also reported that lymphocyte counts decreased early after surgery in liver transplant donors, and the subsequent increase in the regulatory T‐cell population correlated with the rate of lethal complications.^23^ Therefore, even if the use of artificial pancreas is limited within 24 hours, it may contribute to the reduction of perioperative complications such as deep SSI following the lymphocyte count reduction. A previous study showed that IIT using an artificial pancreas reduced serum cytokine levels and the rate of surgical site infections,^1^ suggesting that the use of an artificial pancreas could suppress lymphocyte depletion and maintain immune function, resulting in the suppression of severe complications, which is consistent with our results. Furthermore, by suppressing blood glucose fluctuations, none of our patients under the control of an artificial pancreas experienced complications secondary to hypoglycemia.

The limitation of this study was the small number of patients and the lack of investigation of inflammation‐related factors such as serum cytokine levels. Therefore, the causal relationship between suppression of lymphopenia and reduction of complications remains unclear. Based on the results of this study, we will continue to accumulate the number of cases and investigate these parameters to elucidate the mechanism.

In conclusion, the use of an artificial pancreas for perioperative blood glucose control in pancreatectomy maintained lymphocyte counts and decreased the number of serious complications through proper management of blood glucose levels, without hypoglycemia.

## DISCLOSURE

Conflict of Interest: None.
